# Use of an Endobronchial Blocker Where a Double-Lumen Tube Failed to Ventilate: A Case Report of a Distorted Tracheobronchial Anatomy

**DOI:** 10.7759/cureus.32047

**Published:** 2022-11-30

**Authors:** Muhammad Ammar Amjad, Ahsun Khan, Ali Raza Khan

**Affiliations:** 1 Department of Anaesthesia and Critical Care, Shaukat Khanum Memorial Cancer Hospital and Research Centre, Lahore, PAK; 2 Department of Surgery, Hamid Latif Hospital, Lahore, PAK

**Keywords:** video-assisted thoracic surgery, malignant pleural effusion, one lung ventilation, difficult ventilation, distorted tracheobronchial anatomy, thoracic anesthesiology, double lumen tube, bronchial blocker

## Abstract

One-lung ventilation (OLV) during video-assisted thoracoscopic surgery (VATS) can be accomplished through several different techniques, including bronchial advancement of an endotracheal tube (ETT), use of a double-lumen tube (DLT), or placement of an endobronchial blocker. In most cases, a DLT is a mainstay of isolating and ventilating a single lung during cardiothoracic procedures. The reasons to deploy a DLT over other techniques include ease of placement, less chance of malposition, quick placement time, and quality of lung deflation. However, this case report highlights the importance of a bronchial blocker in a patient where a double-lumen tube failed to ventilate the lungs. Briefly, this young female patient had a right thoracic mass associated with ipsilateral lung collapse and moderate pleural effusion. CT-guided biopsy was planned but was deferred by the radiologist, as the patient was unable to lie in a prone position. The case was then referred to the cardiothoracic surgeon who planned a right VATS and biopsy of the lesion. In the operation theater, after induction of anesthesia, the patient could not be ventilated through a DLT, and high peak airway pressures were encountered. Initially, a size 37 left-sided DLT was used, and subsequently, sizes 35, 32, and 28 were also tried, but all these attempts to ventilate the patient remained futile. A bronchoscopy was done, which did not show any abnormality in the airway. The surgery was postponed due to an inability to ventilate the patient with a double-lumen tube. After a repeat CT scan and draining of 9.3 liters of pleural effusion over a week, the patient was again scheduled for the same procedure but with a changed anesthetic plan. This time around, the anesthetic plan was implemented successfully using a bronchial blocker to isolate the right lung. The surgery went ahead, and the patient had an uneventful postoperative period. The anesthetic management of this patient presented a unique set of challenges, which are shared in this case report.

## Introduction

One-lung ventilation (OLV) during video-assisted thoracoscopic surgery (VATS) can be accomplished through several different techniques [1]. The most common method to isolate a lung and provide optimal thoracic access involves the use of a double-lumen endotracheal tube (DLT). Furthermore, one-lung ventilation can be instituted by advancing an endotracheal tube into a mainstem bronchus and turning it into an endobronchial tube. Another novel, yet rarely used technique to achieve one-lung ventilation is through the blockade of a mainstem bronchus to allow lung collapse distal to the occlusion by using a bronchial blocker. In recent times, a double-lumen tube has been by far the most popular choice for such procedures among anesthesiologists for a variety of reasons, including ease of placement, less chance of malposition, quick placement time, and better quality of lung deflation [2]. The following case report, however, highlights the importance of the deployment of a bronchial blocker in a patient where a double-lumen tube failed to ventilate the lungs.

## Case presentation

**Figure 1:**
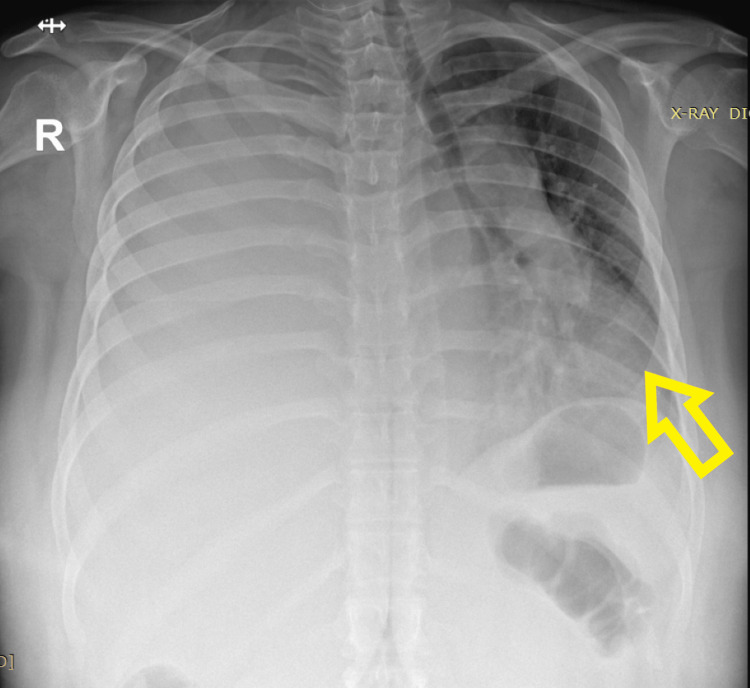
Chest X-ray at presentation Gross right-sided pleural effusion displacing mediastinal contents to the left side. The arrow shows the displaced mediastinal contents and tracheal deviation toward the left side.

A 20-year-old, 160-cm tall female presented with cough and shortness of breath along with persistent right shoulder pain for the last six months. On examination, she had a dull percussion note over the right lung, and auscultation showed decreased breath sounds on the right side. A chest X-ray was done, which showed right lung collapse and right-sided pleural effusion (Figure 1). A computed tomography (CT) scan had already been performed before admission, which revealed an ill-defined mass in the right upper hemithorax measuring 7 x 5.7 cm abutting the posterior thoracic wall along with the aforementioned moderate, right-sided pleural effusion. The case was discussed in a multi-disciplinary team meeting and a CT-guided core biopsy was advised to get a definitive tissue diagnosis. The patient was referred to an interventional radiologist but the radiologist could not perform the procedure because of the inability of the patient to lie prone for a sustained period of time. A right pleurocentesis was done and 1700 ml of straw-colored pleural fluid was drained, which was later proved to be lymphocytic in nature. On the basis of this cytology report, the oncologist was consulted again, and the patient was referred to the cardiothoracic surgeon for a biopsy. The cardiothoracic surgeon reviewed the patient and advised a right VATS and excision biopsy of the lesion. The preoperative assessment was unremarkable other than a leftward mediastinal shift due to right-sided pleural effusion.

In the operation theater, intravenous induction was done after the institution of standard American Society of Anesthesiologists (ASA) monitoring. The patient was easy to ventilate using a bag-mask with a grade 1 Cormack-Lehane view on a Macintosh laryngoscope. To secure the airway, a size 37 Charriere (Ch) left-sided DLT was used, but it could not be passed beyond the 24 cm mark. Once in situ, high peak airway pressures up to 50 cmH2O were observed and the patient could not be ventilated. A size 7 endotracheal tube (ETT) was then passed, which could easily ventilate the lungs when kept at the 18 cm mark. Whenever the endotracheal tube crossed the 20 cm mark, high peak airway pressures were encountered and the patient could not be ventilated. It was thought that a decrease in the size of the DLT would aid in ventilation and thus a size 35 Ch DLT was attempted, but similar to the previous DLT, it could not be passed beyond the 24 cm mark and failed to ventilate the patient. Subsequent attempts with size 32 and 28 Ch double-lumen tubes were also unsuccessful. Fiber-optic bronchoscopy was done through the size 7 ETT but no apparent abnormality of the airways was identified. After trying for approximately 90 minutes, surgery was postponed owing to an inability to ventilate with a DLT and failure to isolate the lungs.

**Figure 2:**
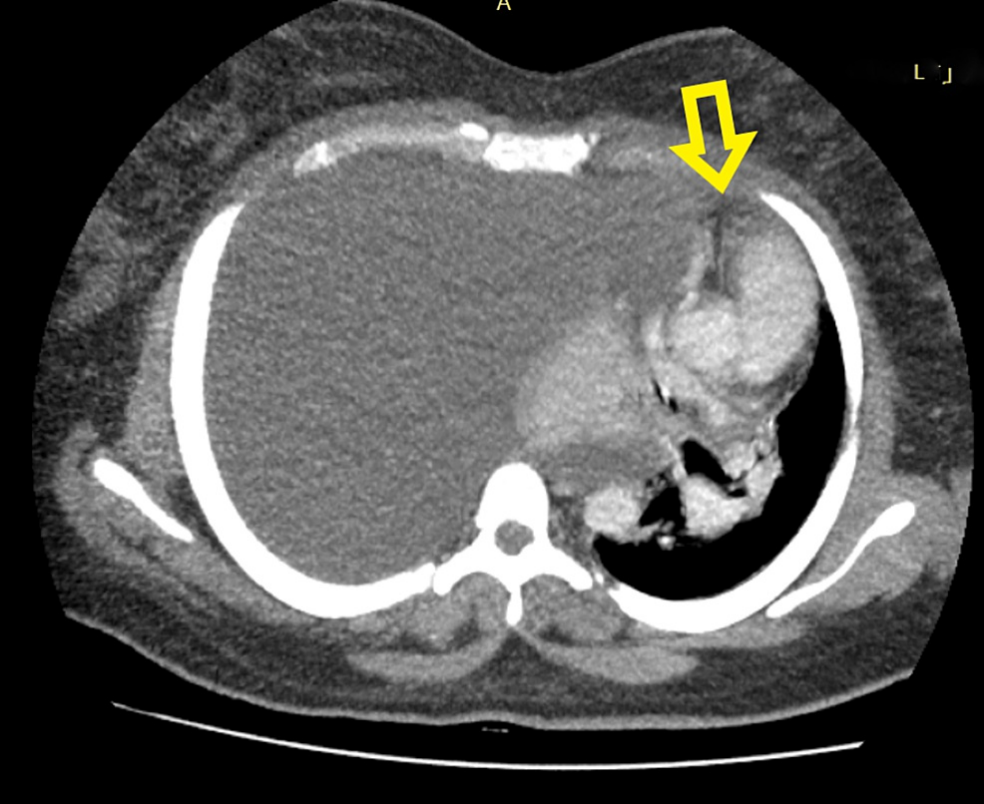
Preoperative CT scan Right hemi-thoracic, pleural-based soft tissue mass with massive pleural effusion causing contralateral mediastinal shift and mass effect on the heart.

Post-procedure, the cardiothoracic surgeon ordered a repeat CT scan (Figure 2), which revealed a re-demonstration of a right, hemi-thoracic, pleural-based soft tissue mass with massive pleural effusion causing contralateral mediastinal shift and mass effect over the superior vena cava, inferior vena cava, and heart. The surgeon advised a 36 Ch chest drain insertion but despite counseling of the patient and the family, chest drain insertion was refused and she got discharged from the hospital. Four days later, she presented again in the emergency department with shortness of breath. An X-ray chest was done, which showed a massive right-sided pleural effusion and mediastinal shift to the contralateral side. She was again counseled for a chest drain insertion and this time around, she did consent to undergo a chest drain placement. A chest drain was inserted and a total of 9.2 liters were drained over the next 7 days. In spite of all these efforts, the patient still did not have a definitive diagnosis, which was essential to commence treatment. Re-exploration was planned by the cardiothoracic team and the patient was planned for a right VATS and biopsy once again. This time around, lung isolation was planned using an Arndt size 9.0 Fr endobronchial blocker with a standard endotracheal tube.

**Figure 3:**
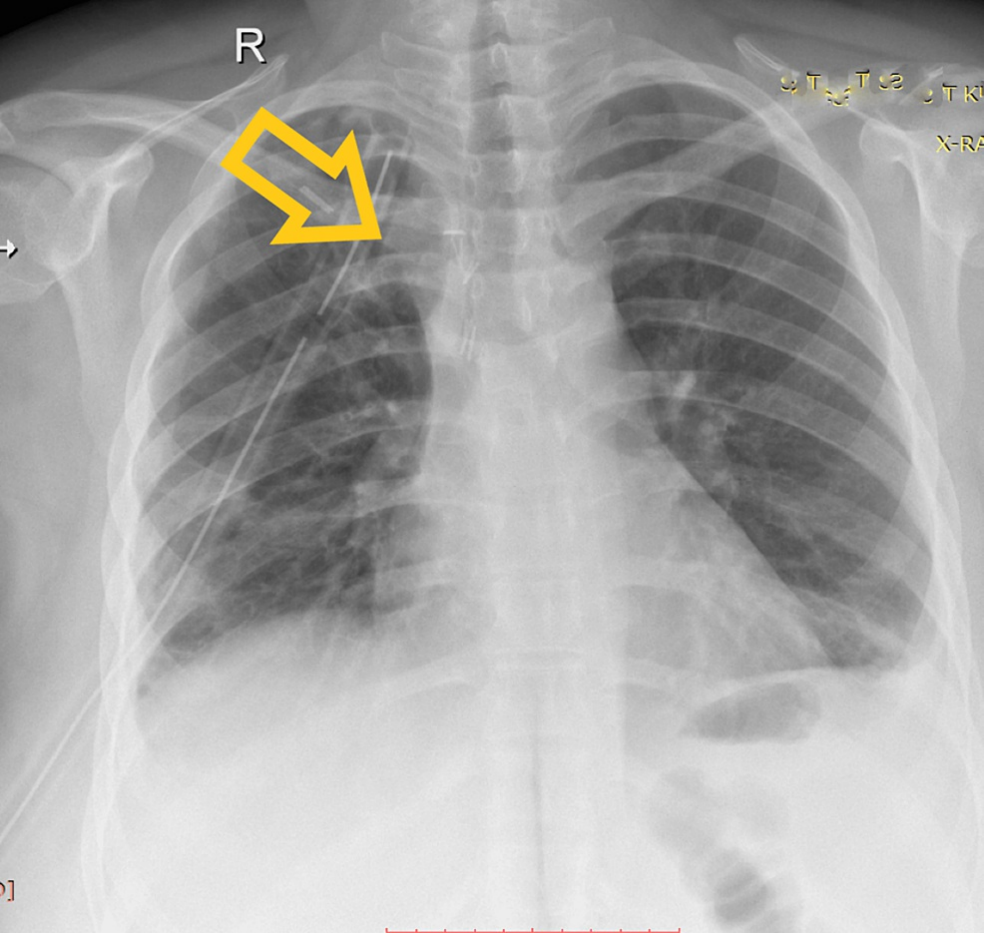
Postoperative chest X-ray The image shows an interval decrease in right-sided pleural effusion. The trachea is back in the midline as well as other mediastinal contents. The right-sided chest tube is in situ.

After standard ASA monitoring was established, anesthesia was induced using midazolam 2 mg, fentanyl 100 mcg, glycopyrrolate 200 mcg, and propofol 100 mg. Muscle relaxation was achieved with atracurium after the ability to ventilate with a bag mask was confirmed. Grade 1 Cormack-Lehane view on a standard Macintosh blade was obtained on laryngoscopy. The airway was secured with a size 6 ETT, but the patient developed high peak airway pressures after intubation, and there was difficulty in ventilating the patient. This was reinforced by capnography, which did not show any carbon dioxide (CO_2_) trace. The patient was extubated and bag-mask ventilation was attempted but ventilation status could still not be improved. A call for help was made. Two-hand bag-mask ventilation was attempted, which improved the ventilation as confirmed by the chest rise and capnography. A left radial arterial line was secured simultaneously. Another intubation attempt was made with a size 6 ETT and the tube was pulled out to the 18 cm mark at the incisors. The peak airway pressures normalized instantly and the patient could be ventilated easily by keeping the ETT at the 18 cm mark. Fiber-optic bronchoscopy was performed through the ETT to look for any potential causes of airway obstruction. The bronchoscopy revealed that the carina had been pushed superiorly as a result of the distorted anatomy of the thoracic contents and massive pleural effusion in this patient. The cranial displacement of the carina meant that there was little room for progression of the distal end of the ETT once the cuff passed through the vocal cords, and it would override the carina on even the slightest of advancement. The size 6 ETT was then removed and replaced with a size 6.5 ETT. Once again, ventilation was effortless when the tube was kept at 18 cm at the incisors. It was deduced at that juncture that when the ETT was pushed further into the trachea, its distal opening would get occluded by the already displaced carina, leading to high peak airway pressures and difficulty in ventilation. Finally, a size 7 ETT was placed and ventilation was confirmed by keeping it at 18 cm at the incisors. The patient could be ventilated easily with the bag as well as the mechanical ventilator. An Arndt size 9.0 Fr endobronchial blocker was placed in the right mainstem bronchus under the bronchoscopic vision and the right lung was isolated. One-lung ventilation was initiated and the patient shifted to a left lateral position. Bronchoscopy was performed again in the left lateral position to confirm the final placement of the bronchial blocker. The patient remained hemodynamically stable afterward and was prepared for surgery. A total of approximately 3 liters of effusion was suctioned out of the pleural cavity after the incision. Right VATS and excision biopsy of the lesion were done uneventfully and the patient was extubated once she was fully awake in the OR. Patient-controlled analgesia (PCA) with morphine was prescribed as postoperative analgesia. Her postoperative stay included two days in the HDU, and she was discharged home on the sixth postoperative day. The postoperative chest X-ray (Figure 3) showed significant resolution of the pleural effusion and relocation of mediastinal structures to the midline. Her biopsy report revealed a low-grade B cell lymphoma for which she underwent chemotherapy successfully. On follow-up scans, the patient remains disease free to date.

## Discussion

Although DLTs are still the most common apparatus used during lung separation [3], an endobronchial blocker is a fascinating technique and in some specific clinical scenarios, it may be more advantageous than a DLT. DLTs are by far the preferred choice among clinicians for subjects who have normal tracheobronchial anatomy. It has been observed that bronchial blockers can be more effective in isolating a lung in patients who have a difficult airway [4] or a distorted anatomy of the upper and lower airways [5]. In recent literature, the use of a bronchial blocker in a prone position has also been observed [6]. Another rare use of a bronchial blocker has been reported as an aid for a malfunctioned bronchial cuff in a DLT [7]. Moreover, the bronchial blocker has also proved to be a lifesaving tool in fatal bleeds from the respiratory tract [8]. In the aforementioned case, the patient’s central airway was displaced by the tumor itself, and the massive pleural effusion rendered the carina move superiorly and the whole mediastinum toward the left side. The aggregate effect of all these findings contrived towards a failure to ventilate using a DLT. It was later discussed and confirmed with the radiologist that the massive amount of fluid in the pleural cavity had pushed the carina cephalad and distorted the 3D anatomy of the mediastinum and the thoracic cavity. Due to this effect, the ETT was abutting the carina leading to high peak airway pressures and the inability to ventilate the patient. The prompt diagnosis of the problem and the use of an underrated technique resulted in the successful completion of this surgery. This case presented a unique set of challenges that are rare in common practice for the anesthetist, and we hope it provides a fruitful learning experience for the reading audience.

## Conclusions

From the above discussion, it can be concurred that a bronchial blocker is a simple, yet novel equipment that has been shown to redeem itself in cases of dire emergencies. A basic understanding of the principles of a bronchial blocker is essential for its use in such cases. The optimal use of a bronchial blocker necessitates an experienced hand as well as prompt detection of any problem that may arise during the process of isolating a lung. In the end, we would like to emphasize the importance of preparedness in terms of backup options when devising an anesthetic plan for a patient with a difficult airway.
